# Supplementary material for: The Onset of Whole-Body Regeneration in Botryllus schlosseri: Morphological and Molecular Characterization
Source: Front Cell Dev Biol. 2022 Feb 14;10:843775. doi: 10.3389/fcell.2022.843775 (PMC8882763; doi:10.3389/fcell.2022.843775)
Supplement: Supplementary file 1 [file Image5.PDF]

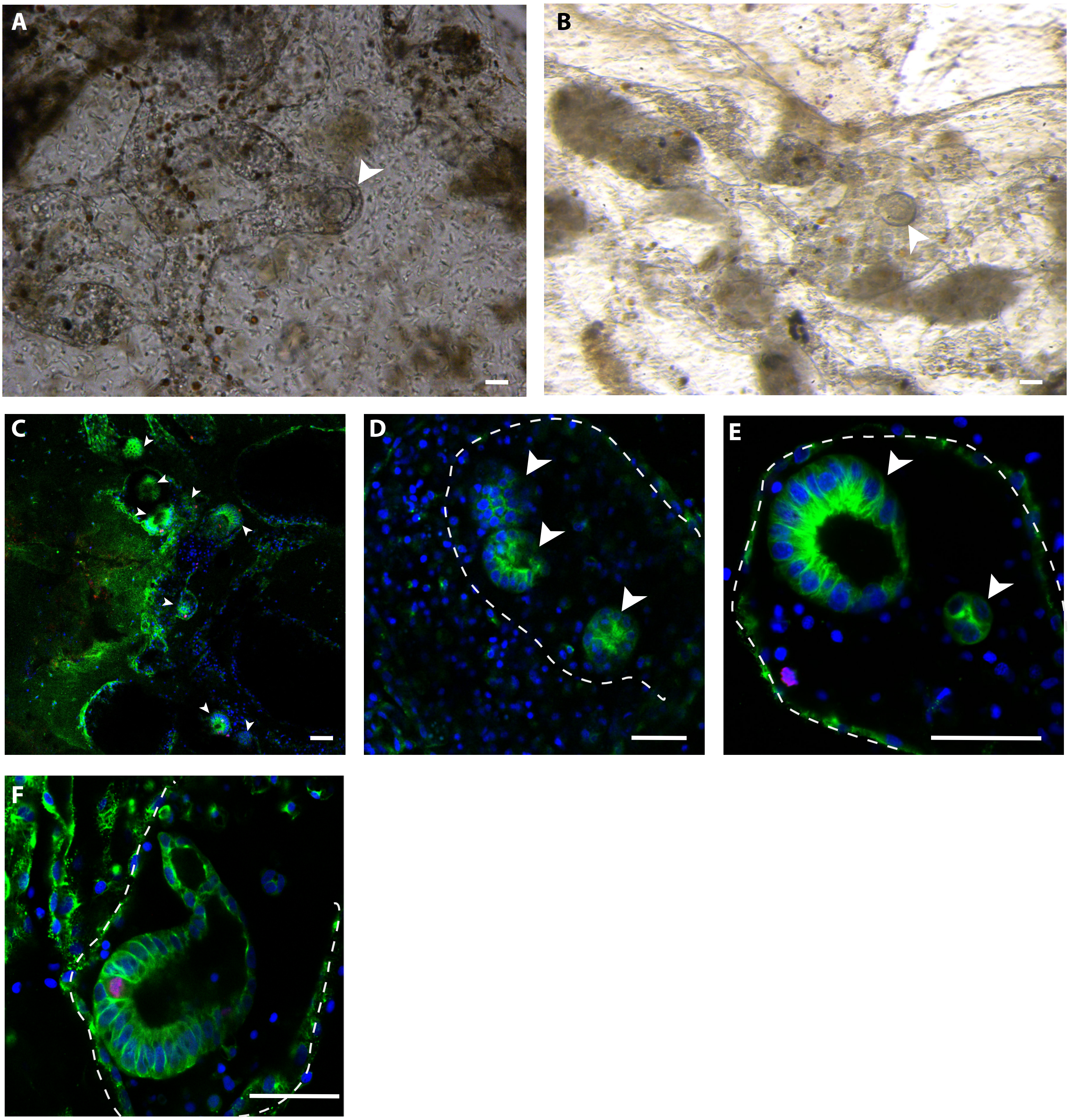

Supplementary figure 5. (A-B) *In vivo* early detection of intravascular vesicles observed withing 3 days after microsurgery. (C-D) Confocal images showing the different structures detected inside the vasculature withing 3 days upon microsurgery, cell shapes are labelled with anti-tyrosinated tubulin (green), proliferating cells are labelled with anti phospho HH3 (red) and cell nuclei are counterstained with Hoescht (blue). (C) presence of numerous monolayered vesicles (arrowheads); (D) details of one ampullae with three monolayered vesicles (arrowheads); (E) detail of polarized monolayered vesicele and cluster of cells (arrowheads) in the tip of a vessel; (F) presence of more complex epithelial structures inside an ampullae. White dotted-line highlight the epithelia of the vessel. Scale bar: 10µ
