# Supplementary material for: The Onset of Whole-Body Regeneration in Botryllus schlosseri: Morphological and Molecular Characterization
Source: Front Cell Dev Biol. 2022 Feb 14;10:843775. doi: 10.3389/fcell.2022.843775 (PMC8882763; doi:10.3389/fcell.2022.843775)
Supplement: Supplementary file 4 [file Image9.PDF]

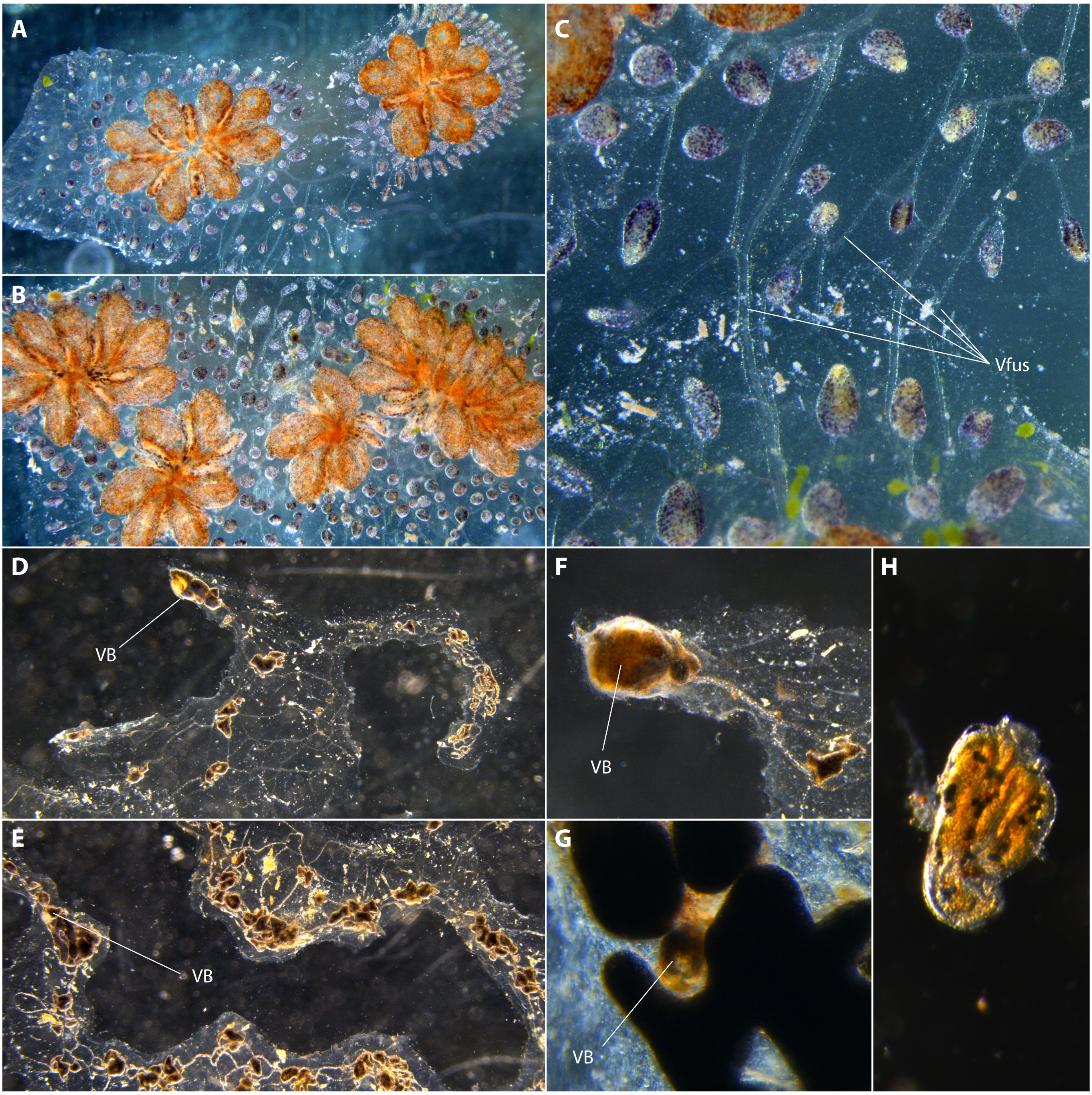

Supplementary figure 9. Sampling of vascular buds from regenerating chimeric colony. (A-B): colonies fused before dissection; (C) detail of the site of fusion between the two colonies; (D and E) regenerating zooid at 13 days after microdisection; (F-G): vascular buds 27 days after microdissection; (H) stomach dissected from a vascular bud (27 days after microdissection). VB = vascular bud; Vfus = fused vessels.
