# Supplementary material for: The Onset of Whole-Body Regeneration in Botryllus schlosseri: Morphological and Molecular Characterization
Source: Front Cell Dev Biol. 2022 Feb 14;10:843775. doi: 10.3389/fcell.2022.843775 (PMC8882763; doi:10.3389/fcell.2022.843775)
Supplement: Supplementary file 5 [file Image10.PDF]

Microsatellite marker	Samples tested	Genotypes	VB genotype correspond to colony genotype
**BS5**	**Colony 1 (C1)**	208 bp and 214 pb	
	**Colony 2 (C2)**	208 bp and 214 pb	
	VB1 in C1	208 bp and 214 pb	
	VB2 in C1	208 bp and 214 pb	
	VB3 in C1	208 bp and 214 pb	
	VB4 in C2	208 bp and 214 pb	
	VB5 in C1	208 bp and 214 pb	
	VB6 in C1	208 bp and 214 pb	
	VB7 in C1	208 bp and 214 pb	
**BS811**	**Colony 1 (C1)**	229 pb	
	**Colony 2 (C2)**	249 pb	
	VB1 in C1	229 pb	YES
	VB2 in C1	N.A	
	VB3 in C1	229 pb	YES
	VB4 in C2	249 pb	YES
	VB5 in C1	229 pb	YES
	VB6 in C1	229 pb	YES
	VB7 in C1	229 pb	YES
**PB41**	**Colony 1 (C1)**	189 pb and 203 pb	
	**Colony 2 (C2)**	200 pb and 203 pb	
	VB1 in C1	189 pb and 203 pb	YES
	VB2 in C1	189 pb and 203 pb	YES
	VB3 in C1	189 pb and 203 pb	YES
	VB4 in C2	200 pb and 203 pb	YES
	VB5 in C1	189 pb and 203 pb	YES
	VB6 in C1	189 pb and 203 pb	YES
	VB7 in C1	189 pb and 203 pb	YES
**PB49**	**Colony 1 (C1)**	240 pb and 242 pb	
	**Colony 2 (C2)**	238 pb and 240 pb	
	VB1 in C1	240 pb and 242 pb	YES
	VB2 in C1	240 pb and 242 pb	YES
	VB3 in C1	240 pb and 242 pb	YES
	VB4 in C2	238 pb and 240 pb	YES
	VB5 in C1	240 pb and 242 pb	YES
	VB6 in C1	240 pb and 242 pb	YES
	VB7 in C1	240 pb and 242 pb	YES

Supplementary Figure 10. Summary table of microsatellite genotyping results. Of 36 fusion experiments between C1 and C2, 7 produced vascular buds (VB1-VB7) harboring either in C1 or in C2. The column “Genotypes” shows the dominant amplicons.
