# Supplementary material for: The Onset of Whole-Body Regeneration in Botryllus schlosseri: Morphological and Molecular Characterization
Source: Front Cell Dev Biol. 2022 Feb 14;10:843775. doi: 10.3389/fcell.2022.843775 (PMC8882763; doi:10.3389/fcell.2022.843775)
Supplement: Supplementary file 6 [file Image6.PDF]

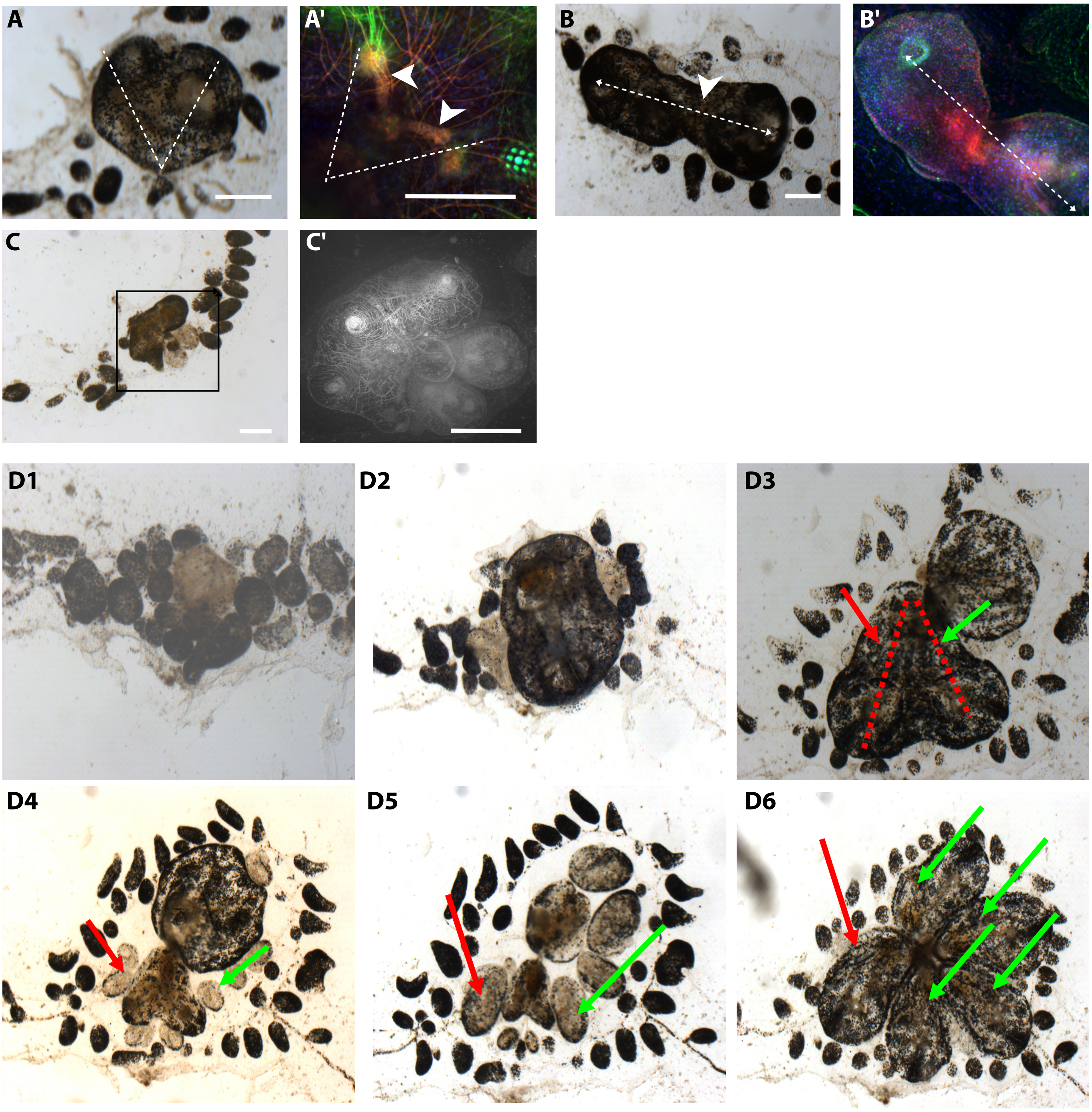

Supplementary figure 6. (A) *In vivo* image of abnormal zooid showing duplication of antero-posterior axis (dotted lines). (A') Confocal image showing the detail of the duplication of the neural gland and ciliated funnel (arrowheads),anti-acetylated tubulin (green), phalloidin (red) and cell nuclei are counterstained with Hoescht (blue). (B) *In vivo* image of abnormal zooid showing posterior fusion (arrowhead) along the anto-posterior axis. (B') Confocal image showing the same abnormal zooid labelled with anti-acetylated tubulin (green), phalloidin (red) and Hoescht (blue). (C) *In vivo* image of abnormal fused zooids. (C') Confocal image showing the same abnormal zooid labelled with phalloidin. (D1-D6) Growth of a vascular bud, followed by asexual budding. (D3) Abnormal zooid showing anterior-posterior axis duplication (red dotted lines): the duplicated hearts (arrows) are inverted. The red arrow shows the correct location of the heart (right side of the zooid) and the green arrows shows the hearts located in the left side of the zooid. Scale bar: 100μ.
