# Supplementary material for: The Onset of Whole-Body Regeneration in Botryllus schlosseri: Morphological and Molecular Characterization
Source: Front Cell Dev Biol. 2022 Feb 14;10:843775. doi: 10.3389/fcell.2022.843775 (PMC8882763; doi:10.3389/fcell.2022.843775)
Supplement: Supplementary file 7 [file Image4.PDF]

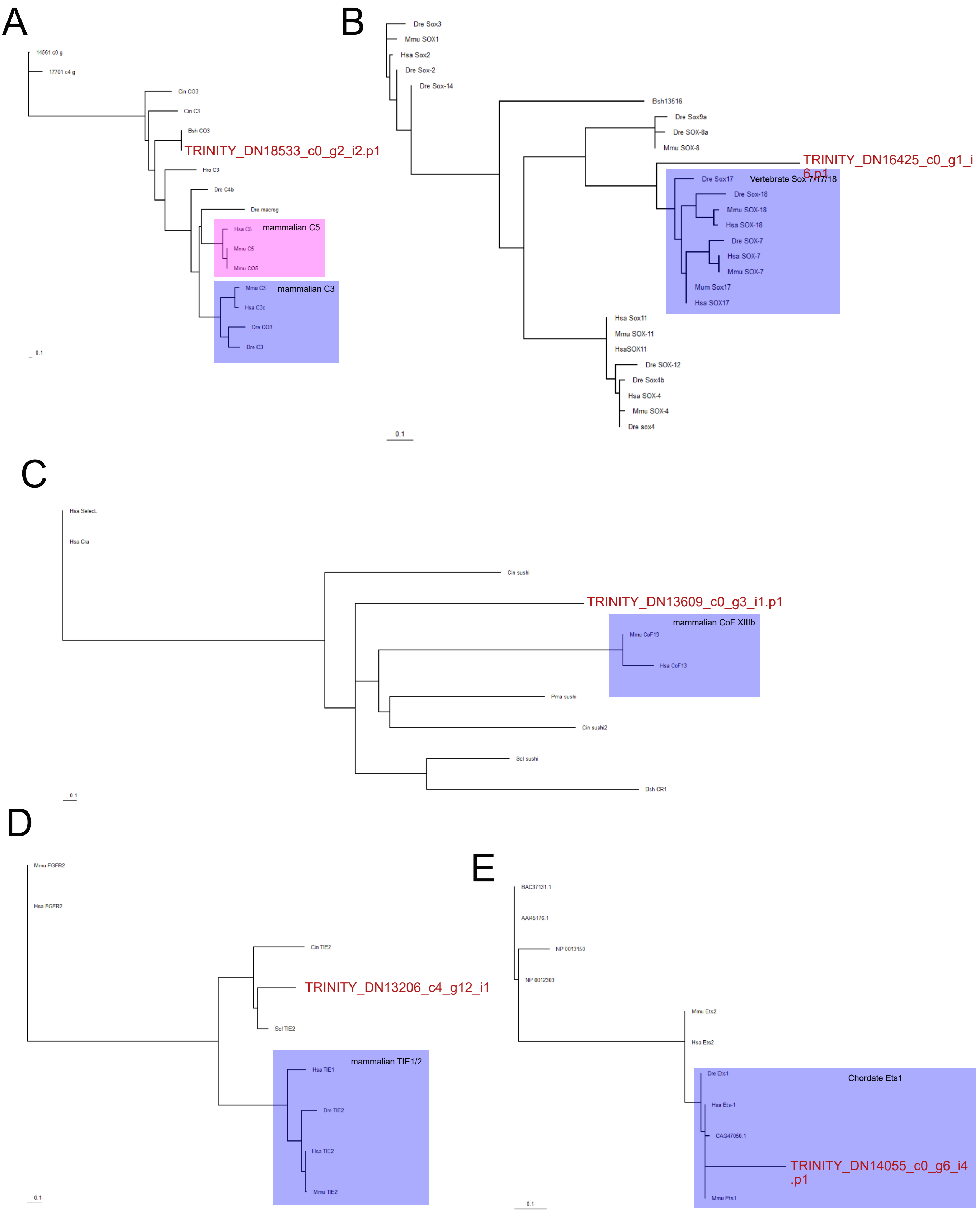

Supplementary Figure 4. **Phylogenetic analyses of genes cited in the results**. (A) Complement C3/5. (B) Sox 7/17/18. (C) Coagulation Factor XIIIb. (D) TIE1/2. (E) Ets-1. Maximum likelihood analyses using PhyML, model LG +G, NNI/SPR mix search. Tree were visually rooted on vertebrate putative paraphyletic outgroup based on blast results. Abbreviations : Bsh, *Botryllus schlosseri* ; Cin, *Ciona intestinalis* ; Dre, *Danio rerio* ; Hro, *Halocynthia roretzi* ; Mmu, *Mus musculus* ; Hsa, *Homo sapiens* ; Pma, *Phallusia mammillata* ; Scl, *Styela clava.* In red : contigs of *Botryllus schlosseri.*
