# Supplementary figures and images for: The Onset of Whole-Body Regeneration in Botryllus schlosseri: Morphological and Molecular Characterization
Source: Front Cell Dev Biol. 2022 Feb 14;10:843775. doi: 10.3389/fcell.2022.843775 (PMC8882763; doi:10.3389/fcell.2022.843775)

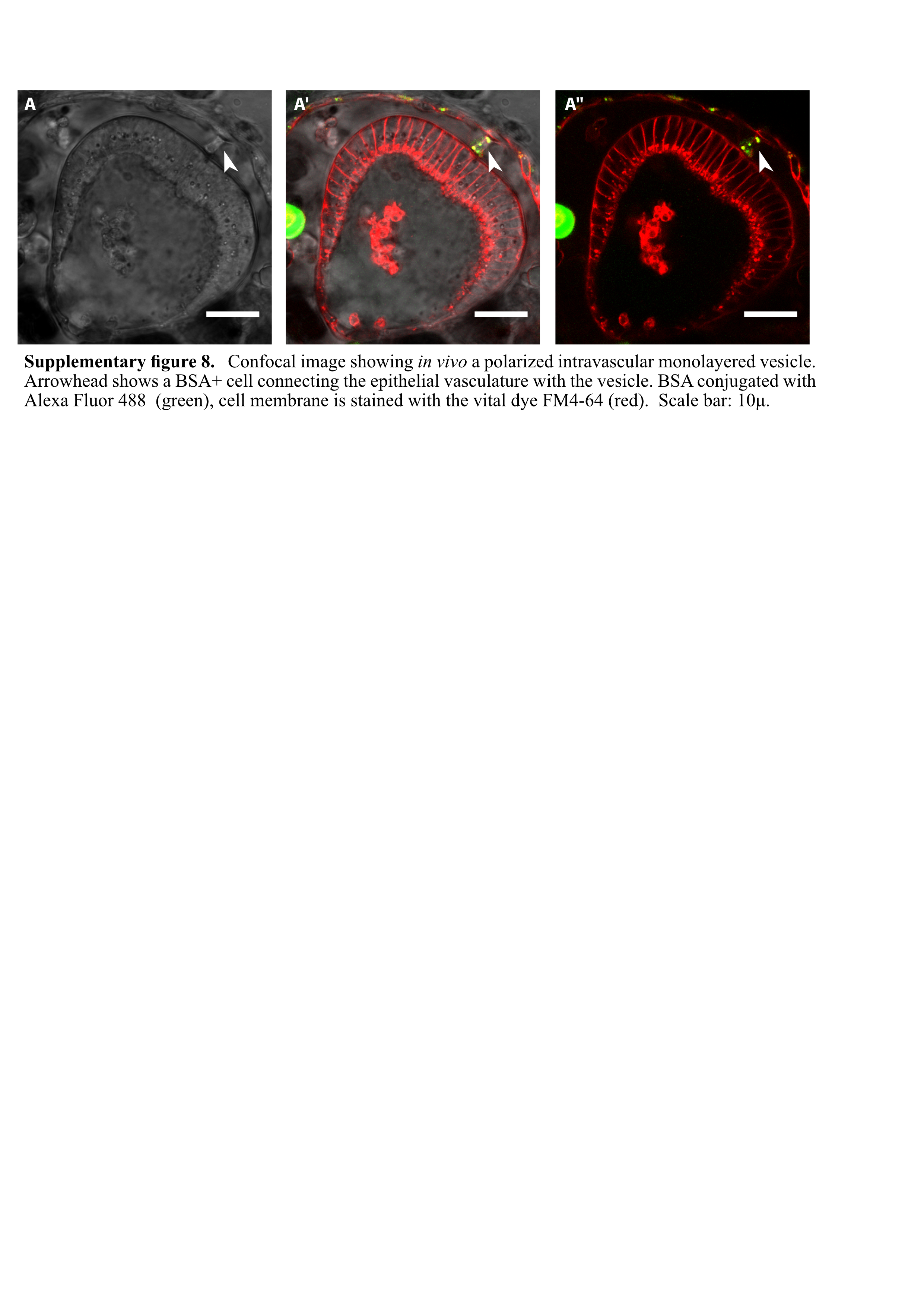

Supplement: Supplementary file 8 [file Image8.TIFF]

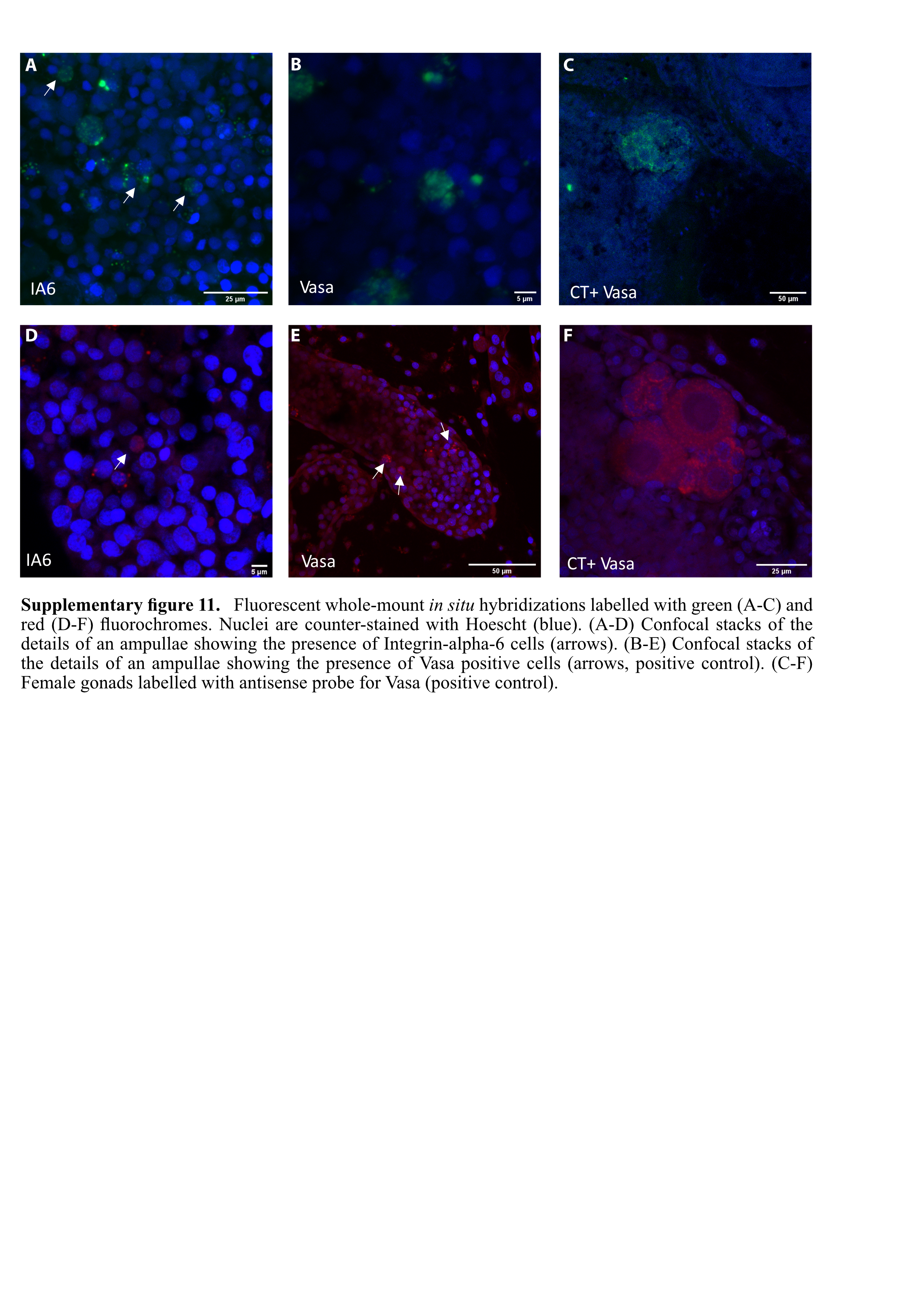

Supplement: Supplementary file 13 [file Image11.TIFF]

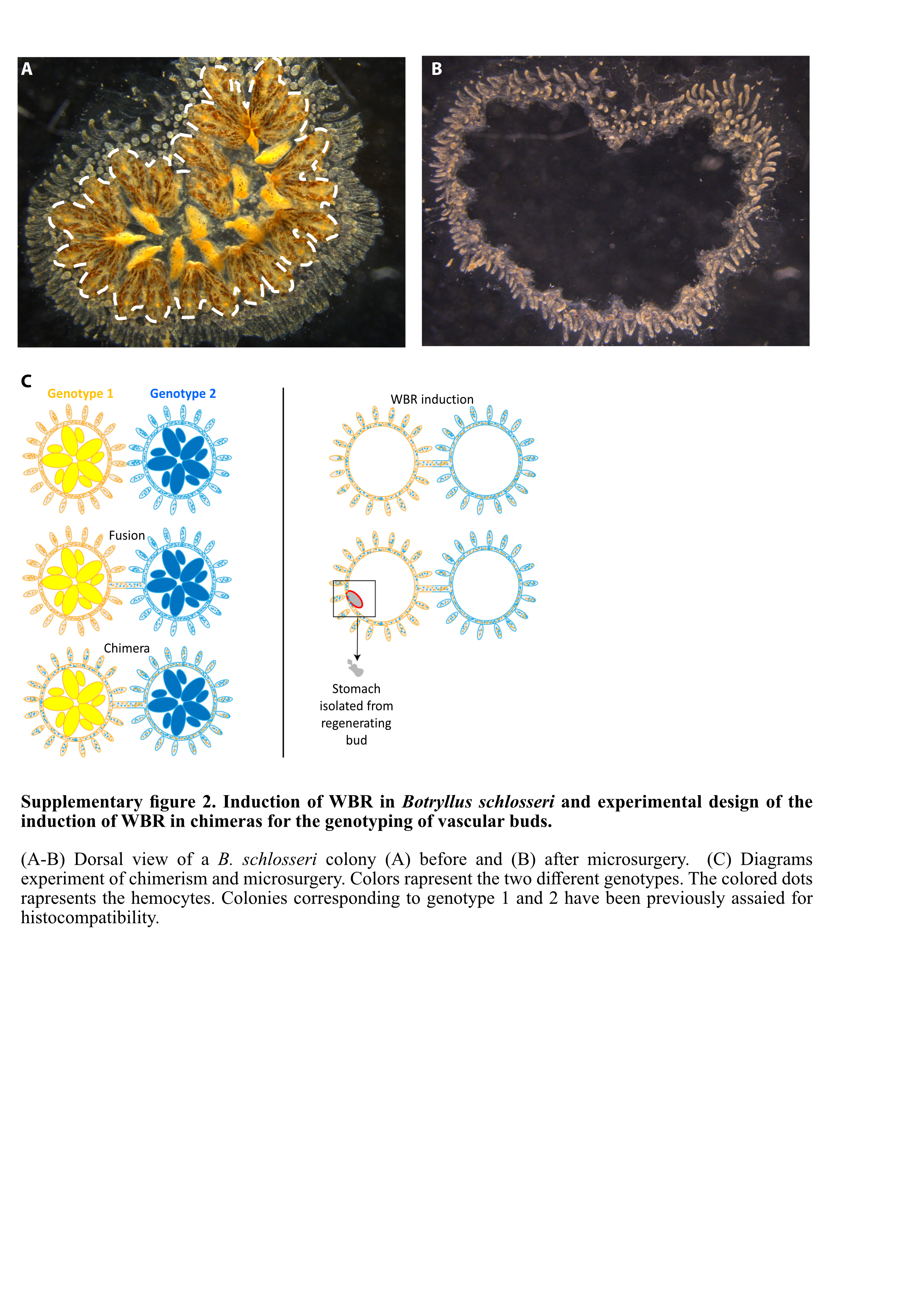

Supplement: Supplementary file 17 [file Image2.TIFF]

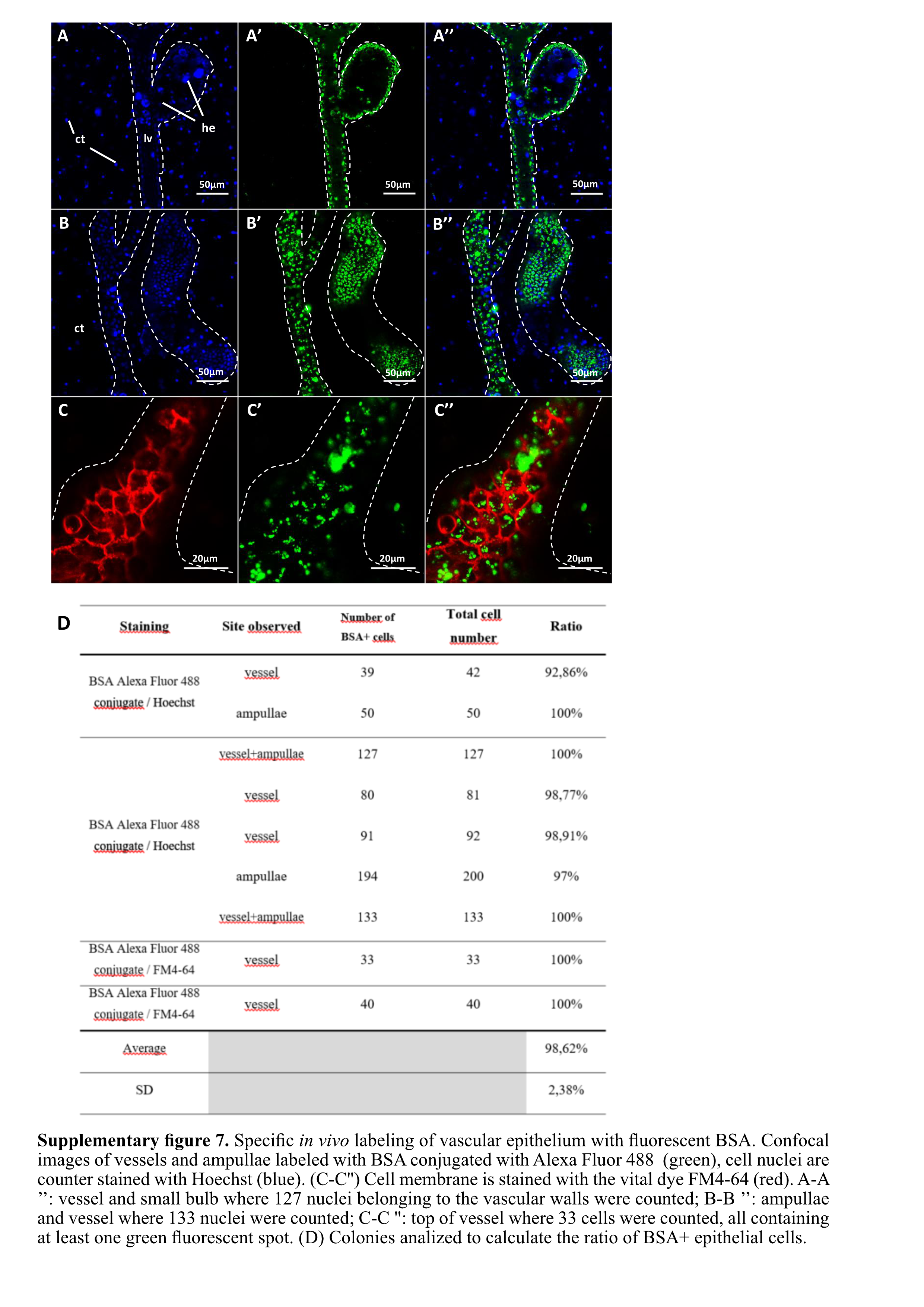

Supplement: Supplementary file 21 [file Image7.TIFF]
