# Supplementary material for: The Onset of Whole-Body Regeneration in Botryllus schlosseri: Morphological and Molecular Characterization
Source: Front Cell Dev Biol. 2022 Feb 14;10:843775. doi: 10.3389/fcell.2022.843775 (PMC8882763; doi:10.3389/fcell.2022.843775)
Supplement: Supplementary file 10 [file Image3.PDF]

WNT2

See: (Di Maio et al., 2015)

Di Maio, A., Setar, L., Tiozzo, S., and De Tomaso, A. W. (2015). Wnt affects symmetry and morphogenesis during post-embryonic development in colonial chordates. *Evodevo* 6, 17. doi:10.1186/s13227-015-0009-3.

INTEGRIN ALPHA-6

>**IA6_v1_Bot_trin2_112712_c0_seq1** len=4477 path=[197006974:0-1273 197013860:1274-1313 197008824:1314-1690 197009303:1691-1709 197009322:1710-1768 197012799:1769-1778 197009391:1779-2020 197013231:2021-2033 197009646:2034-2126 197009739:2127-2148 197009761:2149-2236 197012405:2237-2318 197009931:2319-2689 197012615:2690-2702 197010315:2703-2740 197013300:2741-2750 197010363:2751-3085 197010698:3086-3110 197010723:3111-3448 197011061:3449-3473 197011086:3474-3818 197011431:3819-3876 197011489:3877-4177 197012090:4178-4199 197011812:4200-4201 197011814:4202-4371 197011984:4372-4377 197011990:4378-4408 197012021:4409-4419 197012032:4420-4476]

AGTCTTGTGGTGTTTTGAAATGATAAAAATAAACTGAACTGAACTGATGATGAGATTGCTTGGCGAAACGATT
CCGATTAATGTGTACACTGTGCTACGTCTTCGCCTAGCTAAATCTTGGGGTTGGGCTTCGAATTGAGGTTGGAA
CTTGGAACATTGAACTTGTCAGACGGTCCGCCTTTGTATAGTTAATGGTAATCTGTTGCAGCGTTACGAGACAT
GGGAAATCATAACCCCAGGGGTGCTATTTATTCTATAACTACGGTAATATTCTACAAGAAGTCGCTACTTCAAT
TACTGCTTTTCGTGACCGTTTTAAACTGCTCAGTAGTCAGCTTCAACGTTGAGGAAAGGTATCCTATAGTGAAG
ACAGGACCGACTGGTAGTCTTTTTGGCCTTTCCGTTGCGCAACACCACATCAGGGAAAGCAGCAATGCAAAGG
ACAACGCCATACTACTAGTTGGTGCTCCTACTGATGATCCAGCTAGTGGGGCAGTTGCTCCTGGGCCAGGTGC
ACTTTATAAATGTTCCGTTTTGAACGACGCCAACTGTACCAATGTTCCAATCACACCAAAAGAGGTCCCCGAAG
CGGAAGACATATCGAAGCAGTGGCTTGGTGTGACAGTGAAGTCTTATCGGCCGGGTGGGCGAATTGTGACGT
GCGCTCATCGTTATACTTTGCTCGGGAGGGACTGGGAAGCGCCGCTTGGCCGGTGCTTCATTCTAAAAGCTGA
CTTAACGCCGATTGACGATCGCAATGAGTTCGAATACGTGCCTTGCGAGAATAAGCAGGACACTACCAATACA
TACAGCCATGAGAACTTTGGATATTGCCAGGCTGGCGTTTCCGCTCATTTTGCTGCTGATGTTTCGGGACTGGC
AAACGTGAAGCCATATCTTGTGGTTGGTACTCCCGGCAGCGTACACTGGGGCGGCTCTCTCTCAGCAAAGCGA
CAAATTGGGAATTTCGGTCTGACTATTGAAGATTTATGGTCGAAGCCAAATACAGTTTCGACAAACTTTCAAAT
GGGCACATCTGCTTCGTCTGGGCCGATATACAAGAGTAACGTGCTCAATTTCGTGACTGGCGCGCCCGGCGCA
AACGCCACCGGCAATGTGTACATCTTCGAGGCGAAGTATGTCGGCAGTACTCCGGGCGACGAAAACGATTACA
TGAACGTCCTCGAAGTTCTCGAAGGAGATAAAGTGGCCTCGAGATTCGGTCACGACGTCCTGGTCGTGGATGT
GAACGGCGACGGCAAAATGGACCTTGTTGTTAGCGCGCCGCAATACTATGACAGAAAAGAGCAGCTAGGAGG
CGCCGTTTATGTCTACGTGAATAAAGGCTTGCCGCAACTCGGGCCAATGGCATCTCAGACGTTGTACGGCAAA
ATAGATTCCTACTTTGGAATGGCTATAGCGAGCGCCGGAGACTTGAACATGGACGGCGTTAACGATATCGTTA
TTGGTGCGCCGGGCGATAACGATCGGACTGGTGCGGTGTATATTTATCATGGCGATAACAGTGCTGAGAGTGT
TGGGTTACATTCAAAACCTACTCAGGTGATAGAAGCCAGCATGTTGCGCCCTCTCGGAAGTAATTTCCGCGGG
CTCGGCTATTCGATCAGCGGCGGACTGGACTTGGACATGAACGGTTATCCGGATTTGGCCGTCGGAAGCCTGT
CGGACTCGGCCGTGATATTCTTCTCGCGACCAGTCGTGAATATCTACGGCACCATCGCCGGCCCTACCAAGAA
GATCGAGCTGACGGAAAGTCCTTCGGAACAAGTGCTCAACATCACGGCGTGCCTGAACTACACTGCCTTGCCG
CTGAGTTTCAACGAACGCGTGGCGGTGGTCGGTTATGTCGTGTTGGACACGGGGCGCGTCGAACAGGGCCTG
TTGTCGAGACTGTCGTTCGTGGGCTCGCCCAGCAAAGAGACGGTGGCGAACAAGAGCTTCACGATGACTCTGT
ATCCTCAGTCGACCGGAAGGATTAAGTGTCGGACCTGGAAGACTTATATGAAGAACGATGTGCAAGACAAGC

TGTCGCCGATTGATTTGAAGTTATCATTCAAAACGCCGGACGCGCTCAAGAAGCGGAAGCGTCGCGACACGAC
CTACAAAACCCCGATACCGGTGATGAACGCGGCCGTCGCGAGCACGGCGTCCGCGCAGCTCGAGTTTGCAAA
ACAATGCGGCAGCGACGAGATCTGCAACAGCAACCTGCTGAAAAAGGCTTACTATCAGGTGCTGAAGAACGG
AAACTGGACTGGCTTGAAAGAACGCGACGGCGAGCCTCTGCTGGTTCTGGGCACCGAAGAGCAGATCGGTAT
GGTCATCGAAATCACAAATTATAAAGGCGAAGACGCGCATCAAGCCACGATGGAAATACTGCTCCCGGACGA
GGTGGCGTACCGAAGGATCGATGTCATCACTGGCAACGCCATTCACTGCGACCCGGACACGAAAAACGCGTC
GCTGGTCGTGTGCCATCTGGGAAATCCGTTTAAAGAGGAGGGTCGCGTCAAGTTCCTGCTGAAAATGACGAA
GACGAACAAGATCAATAAGGCCACGGAATTCGCCGTCAGACTGCAGCTGTTAACGACGAGCCAGCAGATGGA
GCCGGACCCCGGGACGGACTATCGCATCTTTGTCGAGGTCGAAGCGCAGCTGATGCTCGACGGCTATCCGAG
CAAGGAACAGGTCAAGTTTTCGGGAGAAGTTGTGGGCAGCGCGGCCGTCAAAAATCCGTCCGACGCCGGGCT
GTTGCACACGCACACGTACGAGGTGTCCAACGCGGGCACCGGCGTCGTAGAAGACGTGGCGTTGAAAATCAG
GTGGCCGCAACAGATCGGGAACGGAAAGTGGCTGTTTTACCTCCTGGAGGCGGACGCCCCGGACCCGGGAAA
CTGTACCCTGCCGAAGAGCGTCAACCCCCTCAGACTGAAGAGCGACGGAAGTCGTCGGAAGCGAGAGGCAGA
TCCCCCGCCCGGAGAATTGAAATCGATTTCCGATTCTGGGACCAGCGATTACGCCATTCTGGACTGCGGTACCG
CCGGTGACGACGTGTCGTGCATGGAGATTACGTGCTTCCTCGGCGATTTGAGGCCGAAATCTACGCAGTCCGT
CAGCTTCACGGCTATACTGTGGAACAGCACGTTTCTCGAGGAGTACAACAGAGTAACCGGGGTCTACGTCACT
TCGTTCGCGGAGCTCACCGTTCTCCAGCAAAACATCAAGTTTTCCGACCACTCCAAGCTGGACAGCGTGGTGGC
GACCACCGTGCTGCCCGAGATCTTGTTTGTGCCGGAAGTGCCGTTGCAGTGGTGGATCATCGTCGTGGCCAGC
GTGGCCGGGCTGTTCCTTCTCGTGTTTCTGATCTTGATCATGTGGAAGTGCGGCTTCTTCAAACGTCAGAAGCA
CGTCGACTATCAGAAGGCGCGGAAACACAAGCAGGCGTCGAAAAAAGCAGAGGAAAAAGAAATATTATACTA
ACTGGATACGGGCGGGCTCAGTCTCACGGAAGTTTACGTCCTAATCACAAGGGCCTTGCGACGTTAGGGCCTG
TTCTCGCTGATAGATTTACGCTACATATCAACGTGTCATCAGTGTTGTTGTAAGAGAGGGCGGTTTGCGTCGTT
CACTGTATTTTGGTATTGCCTTTGATATTGGTGGTTGCTGGTTTGGCCGCTTATCTCGATTGTATAGTTTTGTGC
CGTTAATTCTACCGGGCGGGATAAGCTTGCCCGGCCAGCAGCTTGCGAAAGGATGAAGTTCACAAGTGACACT
TACTAACTACAAACATATCAGTGATTCATCGGCGCGCCGCGATGCGTTGGCAGCTTGAACGGGCCCTTGTCGA
TGTGGTCTGCCGTGTTTTTAACGTTGTAAGAGTCGATTTTTTATCTGTAATACTGTTTTTGTTGTTTGCAAATCGA
TCGAGTGTATTTATCCGTCGTGGTCTTTTTAAACTTTGCTGCGGTGCTGAAAATAGCTCAATTTTATGCATTTTTT
TCTATGACCGTGAGTTTGCTCTTACTCTTACTGCCTTCATATATGACGATAATAATTAATAATGTATCTAATCGGT
TAACATTGCTCTTATACTAAATTGAATCAAGTTCGGTGCTGCTTGCTATACTTATTACGTACAAAGTTTGGTACA
TTTTGTTCTCTGCAGACGGTGGTCAAAATCTGTCTGCCCGCCGACATACACAGCATATATCCCTTGCGTTGGTTC
TGCGGGAGGTCTGAGACCAAATTTTGAGTGTTACTCGATCATGTTACTTCGTAATGCATGGTACCTTTTTGGGC
TTTTGTGGTATTTACGTATTTCATATTCTATTTGCTTTTGCATGATTTACTGAATCGAGTTGTTTTTACGCTTTCAA
GATTTCCTGGTAACAGCGGTTCACGACACAGATACAGTAGGTAATAAAACACCTTCGACTATGAAAAAAAAAA
AAAAAAAAAA

FORWARD PRIMER: GGCTCAGTCTCACGGAAGTT

REVERSE PRIMER: AGATTTTGACCACCGTCTGC

PRODUCT SIZE: 688

Ia6_F1 GGCTCAGTCTCACGGAAGTT

Ia6_R1 AGATTTTGACCACCGTCTGC

VASA

>**VASA_Bot_trin2_108487_c0_seq1** len=2125 path=[160370378:0-480 160371014:481-522 160371082:523-584 160374327:585-596 160373556:597-603 160373563:604-617 160371204:618-676 160371289:677-693 160371319:694-890 160373475:891-948 160371654:949-1106 160371865:1107-1119 160371878:1120-1256 160372043:1257-1281 160372089:1282-1369 160372236:1370-1383 160372250:1384-1499 160372395:1500-1515 160372422:1516-2049 160373118:2050-2124]

TTTTTTTTTTTTTTTCATATACAGCAGAAATGTATCAACGAAACTCAACAAAGATCAGACAAGAGTGCAGCAAG
AAACAAAATCATTCGAGGGCGGCCGCCATAAACGCGACCTCGAGAAACATAATGTGCAGGAACGTTCGATGG
AACACACGGAATAATAAGGCGACAGTGGCATGAGCACCGGAACCGTCAAGCAATACACGCGTTAAGGACACT
ACAATTGCACTAATGAGTGAGCGCCCATTCAATTCCCCCATAGCGACTGTTAGACTTAATAGCCAAGCAGTATG
TGGTACTGATACCTAGGCACTACACACTGCAAAGTAACAACACGTCAAGAAACCGTGCAAAAACCAGCGGCGC
GGCTAAACGAAATACGAGTTCACATCGGATAACAGGATATCCAAGTTCACAATATTTAATTCCACTCATCATCG
CTAAATCCGCCAGAAGCACCACCTCCTTGAGGCGCATTACCAGCCGAGGTCCCGCCATCGTTATAATCATAATC
ACCCGTCTCGCGGTTGGACCTGCCCCCAGACGCCCCGGTAGTCCTGGCGTCTCTAGCACCAAAGCGACCGCCTT
TCGGACCGTGATGCGTGCCCACAGCGCCCAACGCGCAGGTCTCCAACCAATCGGGCACTTCCTGCTGCGCGTC
GGAAAGCACCTTCACCAGCGACCTAGCCAAAGTACCGTCCTTGGAATCGTCGTAAAACGAGGTAGCGGTACCC
AAATTTCCACAACGACCCGTTCTGCCGATTCGATGCACGTACTCGTCAATTTCCGACGGCAAATCGTAGTTGAT
AACGTGCTCCACTTTAGGAATATCAAGGCCTCTAGCTGCCACAGACGTGGCGATCAGTATAGGACAAGTCCCG
TTTTTGAAATCGACCAGAGCCATTTCTCTTTCTCTCTGCTGTCGATCTCCGTGTATACTAGTAGTAGGTAAATTCT
CTTGAGACAGGAGCGTAGCCAGAAAGTCTGCGTTCCTTTTGGTGTCCACAAAGACCAGAGTGCGAGCTTTGGT
TGTCGGAACATCACTCAAAAGCTCCATCAACTTGTCCCTTTTCTCGCTGTGATCAATTTGAATCATAGCCTGAGT
CACATCGGAACAGGCGCCACCGACGCGTCCCACCGTCAAAAACAAAAAGTCGTCTCGCAGAAACTCGTGCGCT
AGCTTTTGTATGTCATCCGGAAACGTAGCGCTGAACATGAGAGTATGCCTCGTGTTTTTATCAGGCATTCCGGG
AGCTCCCAGCAAACGCCTGATCTCCGGTTCAAACCCCATGTCCAACATACGGTCTGCTTCGTCCAAGATGATAT
ATTCGACGCATTCGCAGCTGACTTTACCGCGGTCGATAAAATCCAGTAGCCTACCCGGAGTCGCGATCAACAT
GTTACAACCACGGCTTAAATTAGCCAGCTGGCTGCGTACAGACGTGCCGCCGTAAGCCACCACGGGCTGAATC
ATAGTGCCTCTCGCAAATTTTCTAGCTTCAATGTAGATTTGATTGACCAGCTCTCTGGTAGGCCCGACTACGATG
GCTTGCGGCGTCTGACTCTCGGAAAAAGAATCTGCGGATTTCAACCCGTTACGGAACATGCCGGACAACACGG
GAAGTAAAAAGGCAGCAGTCTTGCCGGATCCAGTCTGCGCGCACGCCATCAAATCCCGGTTATTGTTGACGAT
CGGAATACTGTACTTTTGCACCGGAGTAGGCTTTTCGTACCCAGATTTTAGCACGTTTTGACCGACAGTATCAG
GTAACTTTGCATCGTCGAACGAAGTGATCGGATTCGGCCAGTTTTCGCCAGACACTTCCACTTTGATAGCGTCA
TACTTGTTGAAATTAATTCCAGTCTGTGTAGACGCGTAAATCAGTTCTTCATCCTCAGGGGGCGGTGGTGGTAT
GTAAGGAGGCGGCCTGTCATCATTATCACCGCCCGCGCCACCGTTAGGACACTCCCTCGACATATGCCCTTCTT
CTCCGCACTTGAAACACCCTTTAGGTCTGGAATCGCCTCCTCCGCCGGTCGGGCATTCCCGGGACATATGCCCC
TCTTCTCCACACTTGAAACATCCCTTGGGTCTAGAGTCACCCCCTCCGTTGGGACACTCTCTA

FORWARD PRIMER: GGAGGGGGTGACTCTAGACC

REVERSE PRIMER: ACCACGGGCTGAATCATAGT

PRODUCT SIZE: 653
