# Supplementary material for: The Onset of Whole-Body Regeneration in Botryllus schlosseri: Morphological and Molecular Characterization
Source: Front Cell Dev Biol. 2022 Feb 14;10:843775. doi: 10.3389/fcell.2022.843775 (PMC8882763; doi:10.3389/fcell.2022.843775)
Supplement: Supplementary file 22 [file Image1.PDF]

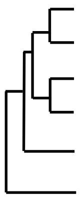

	PROPAGATIVE BUDDING		SURVIVAL BUDDING	
	Peribranchial budding	Vascular budding	Peribranchial budding	Vascular budding
Botryllus primigenus	X	X	-	?
Botrylloides violaceous	X	-	-	X
Botrylloides leachi	X	-	-	X
Botrylloides diegensis	X	-	-	X
Botryllus schlosseri	X	-	-	X
Symplegma brakenhielmi	X	X	-	?

Supplementary Figure 1. Mode of budding in the five species of Botryllidae. The budding has been classified according to Nakauchi (1982) as part of the asexual life-cycle (propagative) or as a response to injury (survival), as well as according to the tissue origin and ontogenesis. The phylogenetic relationships among species have been obtained from Salonna et al. 2021. The presence of survival vascular budding in *B.schlosseri* (red X) is discussed in the present manuscript.

Nakauchi, M. (1982). Asexual Development of Ascidians : Its Biological Significance, Diversity, and Morphogenesis. *Am. Zool.*, 753–763.

Salonna, M., Gasparini, F., Huchon, D., Montesanto, F., Haddas-Sasson, M., Ekins, M., et al. (2021). An elongated COI fragment to discriminate botryllid species and as an improved ascidian DNA barcode. *Sci. Rep.* 11. doi:10.1038/S41598-021-83127-X.
